# VIGE: virus-induced genome editing for improving abiotic and biotic stress traits in plants

**DOI:** 10.1007/s44154-021-00026-x

**Published:** 2022-01-07

**Authors:** Irene N. Gentzel, Erik W. Ohlson, Margaret G. Redinbaugh, Guo-Liang Wang

**Affiliations:** 1 Department of Plant Pathology, The Ohio State University, Columbus, OH 43210 USA; 2 USDA, Agricultural Research Service, Corn, Soybean and Wheat Quality Research Unit, Wooster, OH 44691 USA

**Keywords:** Biotic stress, Abiotic stress, CRISPR/Cas9, Plants, Virus, Gene editing

## Abstract

Agricultural production is hampered by disease, pests, and environmental stresses. To minimize yield loss, it is important to develop crop cultivars with resistance or tolerance to their respective biotic and abiotic constraints. Transformation techniques are not optimized for many species and desirable cultivars may not be amenable to genetic transformation, necessitating inferior cultivar usage and time-consuming introgression through backcrossing to the preferred variety. Overcoming these limitations will greatly facilitate the development of disease, insect, and abiotic stress tolerant crops. One such avenue for rapid crop improvement is the development of viral systems to deliver CRISPR/Cas-based genome editing technology to plants to generate targeted beneficial mutations. Viral delivery of genomic editing constructs can theoretically be applied to span the entire host range of the virus utilized, circumventing the challenges associated with traditional transformation and breeding techniques. Here we explore the types of viruses that have been optimized for CRISPR/Cas9 delivery, the phenotypic outcomes achieved in recent studies, and discuss the future potential of this rapidly advancing technology.

## Introduction

### Plant disease, insect, and abiotic stresses – worldwide impacts

It is estimated that plant disease induces global yields losses between 20 and 40% for rice, maize, wheat, potato, and soybean, five of the most important crops worldwide (Savary et al. 2019). Similarly, global total crop losses due to insect pests are estimated at 18–20% (Sharma et al. 2017). Disease incidence is particularly devastating in countries in which subsistence farms suffer from the compounding effects of poor soil nutrient availability, extreme weather variability, and lack of agricultural resources and infrastructure. As one example, in sub-Saharan Africa, the viral disease maize lethal necrosis (MLN) may cause 100% maize yield loss and production losses nearing or exceeding $300 million USD annually (Pratt et al. 2017; Redinbaugh and Stewart 2018). Aside from disease, environmental stresses, such as drought, contribute to nearly 20% of crop losses worldwide and soil salinity reduces yields to 20–50% of their potential (Leng and Hall 2019; Shrivastava and Kumar 2015). Advances in crop improvement through traditional breeding and/or genetic engineering, paired with conservation practices are of great importance for ensuring a viable agricultural future for nations struggling to achieve global average yields (Shiferaw et al. 2011; Varshney et al. 2021).

CRISPR/Cas9, or clustered regularly interspaced short palindrome repeats/CRISPR-associated protein 9, is a rapidly advancing genomic editing system that provides some advantages over traditional breeding methods, including expediated development time, increased location specificity of the mutation, and the ease of design and implementation of the system (Borrelli et al. 2018). The Cas9 nuclease isolated from the human bacterial pathogen *Streptococcus pyogenes* is the most popular form of Cas9 protein, and has been codon-optimized for use in many plants including maize and soybean (El-Mounadi et al. 2020; Le Rhun et al. 2019; Zhang et al. 2019). Alterations to plant genomes are generated by targeting the Cas9 nuclease to specific sites in the DNA via guide RNAs (gRNAs) (El-Mounadi et al. 2020). Upon nucleotide cleavage by Cas9 to make double stranded breaks, mutations are introduced by error-prone endogenous DNA repair mechanisms. Repair by nonhomologous end joining can introduce base edits, deletions, and gene insertions when donor template is present (Chen et al. 2019; Zhang et al. 2019). High-fidelity homology-directed repair results in gene insertions or deletions based on precise homologous recombination with donor template (Chen et al. 2019; Zhang et al. 2019). Furthermore, multiplexing gRNAs into a single construct allows multiple genes to be targeted during one transformation event (Uranga et al. 2021a). In addition to Cas9 editing, manipulation of gene expression can be accomplished by utilizing deactivated Cas9 (dCas9) derivatives fused to transcriptional activators or repressors (Li et al. 2017; Lowder et al. 2015).

CRISPR/Cas9 constructs can be delivered to plants in several ways, including protoplast electroporation, leaf biolistic bombardment, or leaf infiltrations with Agrobacterium strains carrying the constructs (Varanda et al. 2021). The drawbacks of these methods include the cost of specialized equipment for biolistics, expertise in generating and maintaining viable protoplasts, and difficulty efficiently infiltrating certain species of plant leaves. As an alternative to these traditional methods, new virus-based tools have been developed to deliver CRISPR/Cas9 constructs to plants (Ariga et al. 2020; Varanda et al. 2021; Zhang et al. 2019). Much like other virus-based technologies for protein expression or virus-induced-gene-silencing (VIGS), viral delivery of CRISPR/Cas9 components can greatly enhance functional genomic studies geared towards the development of improved crop varieties, by circumventing the challenges of poor transformation efficiency found among crop species (Ariga et al. 2020; Wang et al. 2020). In this review, we summarize the progress made towards engineering plant viruses into CRISPR/Cas9 delivery constructs and their potential for plant stress resistance research. While many of the studies presented here show the utility of plant viruses to mediate genome editing, we additionally discuss the future development of these virus-induced genome editing (VIGE) vectors to also transiently up- or down-regulate target gene expression.

### Advantages of viral delivery of CRISPR/Cas9 constructs

Plant viruses have contributed to plant genomic studies for decades (Dommes et al. 2019; Wang et al. 2020). In particular, they have been modified for virus-induced gene silencing (VIGS) of plant genes, virus-mediated overexpression (VOX) of heterologous proteins *in planta*, and host-induced gene silencing (HIGS) of trans-species genes (Dommes et al. 2019; Lee et al. 2012; Nowara et al. 2010; Wang et al. 2020). The transient nature of these three distinct molecular biology tools has greatly facilitated plant gene function studies by obviating the need for the time-consuming process of producing transgenic plants (Lee et al. 2012). Additionally, the development of these high-throughput tools has shed light on the limitations and capacities of the viruses themselves (Shi et al. 2021; Wang et al. 2020). Their emerging role as VIGE vectors adds yet another function to their repertoire for CRISPR/Cas9 mediated modification of both model and non-model plants for gene function studies and crop improvement (Wang et al. 2020).

Overcoming problems with poor plant transformation efficiency makes viral delivery of CRISPR/Cas9 constructs desirable by facilitating the use of plants with preferred genetic backgrounds (Ariga et al. 2020). Unlike biolistic methods of Cas9/gRNA construct delivery, viruses replicate *in planta* thereby continuing to increase gRNA titer and thus promoting greater editing efficiency (Hu et al. 2019a). If the infected plants express the Cas9 construct, backcrossing to wild type plants is necessary to obtain filial generations of edited, transgene-free plants (Li et al. 2021). This can be avoided if the complete Cas9/gRNA complex is delivered by the virus to wild type plants, as the plant genome will only contain the CRISPR/Cas9-mediated edits (Ma et al. 2020). This, provided that the virus is not transmitted through seed, alleviates regulatory burdens commonly associated with transgenic organisms.

There are, however, some important considerations when developing viral vectors for this purpose: 1) virus host range and tissue specificity or exclusion (e.g., epidermal, vascular, meristematic tissues); 2) genomic cargo carrying capacity of the virus; 3) mode and efficiency of transmission (mechanical, insect, etc.), and 4) biosafety and biocontainment of the engineered viruses. The host range of a given virus tends to be restricted to either dicots or monocots, although some monocot viruses have been successfully propagated in dicotyledonous species, particularly *Nicotiana benthamiana* (Ellison et al. 2020). Other viruses, such as tobacco rattle virus (TRV) or pea early browning virus (PEBV) have broad host ranges that make them good candidates for CRISPR/Cas9 delivery tools (Ali et al. 2018). Tissue-specificity is another important consideration, as some viruses such as potato leafroll virus (PLRV) are phloem-limited (Bendix and Lewis 2018). Restriction of virus movement could negatively impact the robustness of Cas9-mediated editing and therefore any associated phenotypic outcomes as compared to viruses that spread systemically. Additionally, most viruses are excluded from meristematic tissue, necessitating further modification of the infectious clone such as adding mobile elements to the gRNA sequence (Ellison et al. 2020; Lei et al. 2021).

The size limits of foreign inserts are often dictated by the physical structure of the virus. Typically, rod-shaped viruses can incorporate more foreign genomic material than small (ca. 30 nm) icosahedral viruses (Ariga et al. 2020; Xie et al. 2021). Sonchus yellow net rhabdovirus (SYNV), a bullet-shaped or bacilliform virus, can stably carry up to 5 kb of foreign sequence in its genome (Ma et al. 2020; Peng et al. 2021; Wang et al. 2015). Transmission electron microscopy of SYNV virions expressing Cas9 in addition to a GFP gRNA revealed that the virion length increased by about 35% while the width remained similar to wild type SYNV (Ma et al. 2020). Similarly, the rod-shaped beet necrotic yellow vein virus (BNYVV) had an insert capacity of up to 2.6 kb (Jiang et al. 2019). Viruses that tolerate a greater insert load are attractive for studies requiring larger inserts, such as multiplexed gRNAs, stacked VIGS sequences, or gene overexpression (Jiang et al. 2019).

An important consideration for developing and utilizing viruses for Cas9/gRNA delivery is mode of transmission. Some viruses are easily transmissible to plants by mechanical methods such as rub inoculation, while others require direct injection via vascular puncture inoculation (VPI) or insect transmission (Gao et al. 2019; Redinbaugh et al. 2001). As shown in Table 1, several infectious clones across a range of virus genera have been developed into CRISPR/Cas9 delivery vectors. Many of these viruses, such as tobacco rattle virus (TRV), were previously developed as VIGS or protein expression vectors prior to their development for gRNA delivery (Ali et al. 2015a; Liu et al. 2002; Torti et al. 2021). Interestingly, some of these engineered viruses such as foxtail mosaic virus (FoMV), potato virus X (PVX), and TRV, do not have known natural vectors and can be transmitted to plants via biolistics. This is advantageous from a biosafety standpoint, as transmission to test plants in the absence of an insect or bacterial vector provides a higher level of biocontainment to reduce the likelihood of accidental release into the environment (Brewer et al. 2018). Seed transmission of engineered viruses is another biosafety issue, although many of the studies we describe in this review determined that edited progeny plants were virus-free (Brewer et al. 2018).

**Table 1:** Infectious viral vectors for delivering CRISPR/Cas9 and/or gRNAs to plants for genomic editing

Genus^**a**^	Virus Name	Plant(s) Used	Laboratory Inoculation Method(s)	Virus Insert Cargo	Host Gene Target(s)^**c**^ and editing frequency^**d**^	Mutations heritable?	Reference
***Begomovirus***	Cabbage Leaf Curl virus (CaLCuV)	Cas9-overexpressing *N. benthamiana*	Agrobacteria infiltration	Single gRNA	*NbIspH:* 75% *NbPDS:* 85%	Not determined	(Yin et al. 2015)
	Cotton leaf crumple virus (CLCrV)	Cas9-overexpressing *Arabidopsis thaliana*	Agrobacteria infiltration	Single gRNA (+/− FT)	*AtBRI1:* 25–50% *AtGL2:*18.8–62.5%	Yes (4.4–8.8%)	(Lei et al. 2021)
***Benyvirus***	Beet necrotic yellow vein virus (BNYVV)	Cas9-overexpressing *N. benthamiana*	Agrobacteria infiltration	Single gRNA	*NbPDS3:* 85%	Not determined	(Jiang et al. 2019)
***Beta-Nucleo-rhabdovirus***	Sonchus yellow net rhabdovirus (SYNV)	*N. benthamiana (WT or GFP-*expressing*)*	Agrobacteria infiltration, rub inoculation	Cas9 and single or multiplexed gRNAs	*GFP: 77–91%* *NbPDS:* 40–79% *NbRDR6:* 53–91% *NbSGS3:* 79–91% Multiplexed *NbRDR6 + NbSGS3:* 60–96%	90–100% via tissue regeneration; indels maintained in M1 and M2 generations after selfing	(Ma et al. 2020)
***Cyto-rhabdovirus***	Barley yellow striate mosaic virus (BYSMV)	*GFP-*expressing *N. benthamiana*	Agrobacteria infiltration	Cas9 and single gRNA	*GFP:* demonstrated by sequencing but not quantified	Not determined	(Gao et al. 2019)
***Hordeivirus***	Barley stripe mosaic virus (BSMV)	WT or GFP-expressing *N. benthamiana;* Cas9-expressing wheat/maize	Agrobacteria infiltration, rub inoculation	Single or multiplexed gRNAs	*NbPDS:* 19–80% Multiplexed *NbPDS* + GFP: 10–12% *TaGASR7:* 78% *ZmTMS5:* 48%	Plants regenerated from *N. benth* tissue exhibited mutations; not determined for wheat and maize	(Hu et al. 2019a)
		*N. benthamiana;* Cas9-expressing wheat	Agrobacteria infiltration; rub inoculation	Single gRNA (+/− FT)	*TaPDS:* 3.8–96.1% *TaGW2*: > 75% *TaGASR7*: > 70%	Yes: 46–69% in M1, transmitted to M2	(Li et al. 2021)
***Mastrevirus***	Bean yellow dwarf virus (BeYDV)^b^	Tomato	Agrobacteria infiltration	Cas9 and single gRNA	*SlCRTISO:* 90.4% *SlPSY1: 56.4%* Gene replacement: 25%	Gene replacement: Progeny of T_0_ plants segregated for red vs orange fruit color.	(Dahan-Meir et al. 2018)
***Potexvirus***	Potato virus X (PVX)	*N. benthamiana*	Agrobacteria infiltration; rub inoculation	Cas9 and gRNA	*NbTOM1*	62% plants regenerated from infected tissue had *NbTOM1* edits.	(Ariga et al. 2020)
		Cas9-expressing *N. benthamiana*	Agrobacteria infiltration	Single/multiplexed gRNAs +/− tRNA spacers, mobile *FT*	*NbXT2B: 37–85%* *NbPDS: 25–73%* *NbFT: 52%*	46–95% for regenerated plants from infected tissue; germline transmissible with mobile *FT* gRNA modification	(Uranga et al. 2021a)
	Foxtail mosaic virus (FoMV)	Cas9-expressing *N. benthamiana/ S. viridis/ Z. mays*	Agrobacteria infiltration; rub inoculation	Single gRNA	*NbPDS: 73–91%* *SvCA2: 45–60%* *ZmHKT1: 7–38%*	NbPDS: Not heritable SvCA2: Not heritable ZmHKT1: not determined	(Mei et al. 2019)
***Tobamovirus***	Tobacco mosaic virus (TMV)	GFP-expressing *N. benthamiana*	Agrobacteria infiltration	Individual or simultaneous delivery of Cas9 and single or multiplex gRNAs	*GFP: 61–63%* *NbAGO1: 6–27%* *Multiplexed: 11–64%*	Not determined	(Chiong et al. 2021; Cody et al. 2017)
***Tobravirus***	Tobacco rattle virus (TRV)	Cas9-expressing *N. benthamiana /A. thaliana*	Agrobacteria infiltration; rub inoculation	Single gRNAs	*NbPDS3: 21–57%* *NbPCNA: 45–63%* *AtGL1: 21%* *AtTT4: demonstrated by sequencing*	*NbPDS3* edits detected in seed from earliest developing flowers; not determined for Arabidopsis	(Ali et al. 2015a, b, 2018)
		Cas9-expressing *N. benthamiana*	Agrobacteria infiltration	Multiplexed gRNAs with mobile *FT* or tRNA modifications	*NbPDS3: 58%* *NbAG:53–86%* *Multiplexed: 10–95%*	Mutations detected in progeny for two generations.	(Ellison et al. 2020)
	Pea early browning virus (PEBV)	Cas9-expressing *N. benthamiana*	Agrobacteria infiltration, rub inoculation	Single or multiple gRNAs	*NbPDS: 36–72%*	Not determined	(Ali et al. 2018)

^a^Genera data from (Walker et al. 2020) and https://talk.ictvonline.org/files/master-species-lists/m/msl/12314

^b^Now classified as chickpea chlorotic dwarf virus (CpCDV) (Kanakala and Kuria 2018)

^c^Gene abbreviations:

*Arabidopsis thaliana: AtGL1 (GLABRA1); AtTT4 (TRANSPARENT TESTA GLABRA4); AtBRI1 (BR-insensitive 1)*

*Nicotiana benthamiana: NbTOM1 (Tobamovirus multiplication 1); NbXT2B (UDP-xylosyltransferase 2B); NbPDS (Phytoene desaturase); NbFT (Flowering locus T); NbIspH (isopentenyl/dimethylallyl diphosphate synthase); GFP (green fluorescent protein); RDR6 (RNA-dependent RNA Polymerase 6); SGS3 (Suppressor of Gene Silencing 3); NbAGO1 (ARGONAUTE1); NbPCNA (proliferating cell nuclear antigen)*

*Solanum lycopersicum (tomato): CRTISO (carotenoid isomerase) and PSY1 (phytoene synthase 1)*

*Triticum aestivum (*wheat*): TaGASR7 (Gibberellic Acid-Stimulated Regulator 7)*

*Zea mays (maize): ZmTMS5 (thermosensitive genic male-sterile 5); ZmHKT1 (high-affinity potassium transporter 1)*

*Setaria viridis (green millet): SvCA2 (Carbonic anhydrase 2)*

^d^Editing efficiencies (indels) determined by PCR/restriction digest assays of the DNA target region or sequencing unless specified otherwise

### Available virus-mediated CRISPR/Cas9 plant genome editing tools

As summarized in Table 1, numerous viruses have been adapted for delivering the Cas9/gRNA components to plants. Most of these VIGE systems were developed and tested in *N. benthamiana* due to the ease of producing viral inoculum via leaf agroinfiltrations. If the virus system replicates sufficiently in *N. benthamiana*, infected leaves can be harvested to generate inoculum for experiments with other plants that are more difficult to infiltrate. Additionally, stable transgenic Cas9-expressing *N. benthamiana* lines facilitate testing efficiency of gRNAs during development of the viral system. As one example, Jiang et al. (2019) developed a BNYVV system for co-expressing multiple proteins in sugar beet. To further develop this virus for CRISPR/Cas9 editing capabilities, experiments with this system delivering a gRNA targeting *PDS* in Cas9-overexpressing *N. benthamiana* resulted in photobleaching of 78% of the inoculated plants. This suggests that BNYVV may also be a useful genome editing tool in sugar beet once Cas9-expressing plants are available (Jiang et al. 2019).

For some crop plants, stable Cas9 transgenics are available to directly study gene function after viral delivery of gRNAs. While these transgenic plants would require backcrossing to eliminate the Cas9 transgene prior to agricultural use, they are very useful for testing the effectiveness of gRNAs or when the virus of interest has restricted foreign insert capacity. Hu et al. (2019a) demonstrated the gene editing capabilities of barley stripe mosaic virus (BSMV) in *N. benthamiana* as well as wheat and maize. Previously, BSMV had been developed as a protein expression vector, demonstrated by a large 2 kb GFP fusion with an aluminum malate transporter gene, *TaALMT1,* that improved aluminum toxicity tolerance in wheat (Cheuk and Houde 2018). After confirming successful editing by BSMV delivery of *PDS* gRNA into *N. benthamiana* leaves co-infiltrated with Cas9 constructs, Hu et al. (2019a) tested the system with transgenic Cas9-expressing wheat and maize. In wheat, gRNAs targeting the grain length and weight gene, *TaGASR7,* had mutation efficiencies up to 78% as indicated by restriction digest analysis of the target gene. This study did not investigate the phenotypic effects of the *TaGASR7* mutations; however, which will likely be addressed in future studies. In maize plants, gRNAs targeting *thermosensitive genic male-sterile 5 (ZmTMS5)* were determined to have editing efficiencies up to 48% (Hu et al. 2019a). A subsequent study showed that multiple BSMV constructs could be co-inoculated to simultaneously target multiple genes in wheat without concern for superinfection inclusion, which is usually avoided by using a single multiplex construct (Li et al. 2021).

To address concerns of low gRNA expression by viral vectors, Cody et al. (2017) developed a modified tobacco mosaic virus (TMV) vector lacking a coat protein to prevent systemic movement through the plant and thus increase local viral titer for transient expression assays. When GFP gRNAs were co-infiltrated with Cas9, nearly 70% editing efficiency in GFP-overexpressing *N. benthamiana* leaves was observed. Subsequent experiments targeting *ARGONAUTE1* paralogs *NbAGO1-H* and *NbAGO1-L* also resulted in genomic edits, although at lower efficiency (Cody et al. 2017). Although most other CRISPR/Cas9 virus systems are intended to generate heritable mutations, the focus of this TMV system is to provide transient editing technology complementary to established VIGS methods (Cody et al. 2017). A follow-up study further optimized the system using RNA interference suppressors and simultaneously delivering Cas9 and gRNAs from a single TMV construct to eliminate the need for transgenic plants or co-delivery of the components from separate constructs (Chiong et al. 2021). Though editing efficiency was lower when using a single construct compared to co-delivery, it was nonetheless possible to obtain ~ 7% editing efficiency in *N. benthamiana* even with the large insert load of about 4.2 kb (Chiong et al. 2021).

One of the highest editing efficiencies reported to date was obtained using SYNV (Ma et al. 2020). As discussed earlier, SYNV can carry a large insert cargo, making it a good candidate for expressing Cas9 as well as single or multiplexed gRNAs. Ma et al. (2020) demonstrated this in *N. benthamiana*, where editing efficiency ranged from 40 to 91% in plants infected with single gRNA constructs targeting *GFP*, *NbPDS*, *NbRDR6*, or *NbSGS3*. They also observed similar editing efficiency when SYNV constructs contained multiplexed *NbRDR6* and *NbSGS3* gRNAs, indicating further the practical utility of this virus system. While no progeny of the virus infected plants carried mutations, editing efficiencies of 90–100% were obtained in plants regenerated from infected tissue (Ma et al. 2020). Similarly, another rhabdovirus system using barley yellow striate mosaic virus (BYSMV) was developed to express Cas9 and GFP-gRNA, resulting in genomic editing of GFP-expressing *N. benthamiana* (Gao et al. 2019).

Potexviruses have also been used as gRNA delivery vehicles. PVX was successful in delivering Cas9 as well as gRNAs to *N. benthamiana* plants via agroinfiltration (Ariga et al. 2020). Additionally, this group replaced Cas9 with a larger base-editing version, which proved to be stably integrated into the virus genome. While PVX did not infect the germline to produce edited progeny, plants regenerated from rub-inoculated tissue yielded plants with *NbTOM1* edits, although at a lower efficiency compared to those regenerated from agroinfiltrated plants (62%). It was later revealed that PVX could be useful for delivering multiplexed gRNAs, and that edits were heritable when gRNAs included mobile *FT* modifications (Uranga et al. 2021a). Another potexvirus, FoMV, was previously developed as a VIGS vector and protein expression system (Beernink et al. 2021; Liu and Kearney 2010; Mei and Whitham 2018; Mei et al. 2016). The FoMV vector indicates it has potential as a gRNA delivery system for *N. benthamiana*, maize, and green millet (Beernink et al. 2021; Mei et al. 2019). Although editing was observed in both inoculated and systemic tissue including flowers, the edits were not heritable in *N. benthamiana* (Mei et al. 2019). Using the FoMV system to target the maize salt-tolerance gene *ZmHKT1* in segregating Cas9-expressing maize resulted in 3–6% editing efficiency, which increased to 7–38% when plants were co-inoculated with sugarcane mosaic virus (SCMV) to create a synergistically more robust infection (Mei et al. 2019). Even higher editing efficiency was observed for FoMV targeted *SvCA2* of green millet, with 45% in inoculated leaves and 60% in systemic leaves (Mei et al. 2019).

Two tobraviruses, tobacco rattle virus (TRV) and pea early browning virus (PEBV), are currently being used as CRISPR/Cas9 delivery vectors. TRV, with a wide host range and an easily modifiable bipartite positive sense RNA genome, is a proven VIGS vector for *N. benthamiana*, tomato, and more recently maize and wheat (Liu et al. 2002; Senthil-Kumar and Mysore 2014; Zhang et al. 2017). Initial studies showed that TRV could successfully edit *PDS3* and *PCNA* genes in *N. benthamiana*, either singly or simultaneously when gRNAs were co-delivered from separate constructs (Ali et al. 2015a). In that study, germline *PDS3* editing was observed in seeds collected from the earliest developed flowers, obviating plant regeneration from infected tissue (Ali et al. 2015a, b). Further testing of this system in Cas9-expressing Arabidopsis showed that TRV delivery of *AtGLI* or *AtTT4* gRNAs could produce indels at those target sites (Ali et al. 2018). Interestingly, a direct comparison of TRV versus PEBV editing efficiency of *PDS3* in Cas9-expressing *N. benthamiana* showed PEBV had a much higher editing efficiency (27–35% compared to 57–63%, respectively) (Ali et al. 2018). Recently, TRV-delivered gRNAs produced heritable edits when the gRNAs were fused with mobile FT or tRNA sequences targeting *NbPDS* and *NbAG* (Ellison et al. 2020). Another study used TRV gRNA delivery to target viral pathogens directly, rather than focusing on plant defense related gene targets to increase resistance (Ali et al. 2015c). Here, the group reported that gRNAs targeting geminivirus tomato yellow leaf curl virus (TYLCV) resulted in ~ 42% editing efficiency and correlated with symptom reduction in infected Cas9-expressing *N. benthamiana* plants (Ali et al. 2015c).

Most of the CRISPR/Cas9 virus delivery systems to date rely on RNA viruses; however, there are a few DNA viruses – notably geminiviruses – that also have been developed for this purpose (Baltes et al. 2014; Dahan-Meir et al. 2018; Lei et al. 2021; Yin et al. 2015). Cabbage leaf curl virus (CaLCuV) was shown to be an effective VIGS and miRNA expression vector prior to modification for gRNA delivery to Cas9-expressing *N. benthamiana* plants (Yin et al. 2015). Removal of the AL1 insect transmission protein attenuates symptoms but not virulence, a useful characteristic for assessing phenotypes more clearly for genes of interest (Yin et al. 2015). When targeting *PDS*, CaLCuV gRNA delivery resulted in 85% mutation efficiency, which was higher than with gRNAs targeting *NbIspH* (75%) (Yin et al. 2015). Another virus-induced genome editing system developed by Lei et al. (2021) used cotton leaf crumple virus (CLCrV) (Lei et al. 2021). In this example, the study added a mobile FT sequence to the gRNAs for heritable genome editing of *AtBRI1* and *AtGL2* in Arabidopsis - modifications that have also proven effective in other virus systems (Ellison et al. 2020; Lei et al. 2021; Uranga et al. 2021a). In one of the few studies that used agroinfiltration of plants other than *N. benthamiana*, Dahan-Meir et al. (2018) developed bean yellow dwarf virus (BeYDV) replicons into a gRNA delivery vehicle for tomato. Interestingly, they were able to restore red fruit color to a fast-neutron generated *crtiso* deletion mutant (orange fruit) through double stranded break/homologous recombination of the wild type *CRTISO* sequence delivered by the BeYDV-Cas9 construct (Dahan-Meir et al. 2018).

### The future of virus delivery of CRISPR/Cas9 to plants for engineering disease, insect and stress resistance

Traditional transformation methods with CRISPR/Cas9 constructs were used to target a variety of plant phenotypes, including reduced lodging in elite rice cultivars, drought tolerance in Arabidopsis and maize, and pathogen resistance across numerous plant species (Hu et al. 2019b; Nuñez-Muñoz et al. 2021; Varanda et al. 2021; Wang et al. 2019; Zaynab et al. 2020). The diverse panel of CRISPR/Cas9 viral systems discussed in this review – many just recently published as proof-of-concept – have great potential for developing improved crop varieties with resistance to disease, pests, and abiotic stresses. Utilizing viral systems to deploy CRISPR/Cas9 constructs to yield germline edits would save time and resources usually required for regenerating plants, and can eliminate years of backcrossing when the virus system is capable of delivering the full Cas9-gRNA complex. Additionally, many studies have shown that the inoculated virus is not detected in progeny plants unless vegetatively propagated, thus eliminating regulatory concerns. Given that this methodology is still in its infancy, few of the studies we describe here have addressed plant diseases or abiotic stresses. Insect control using CRISPR/Cas9 technology is also very limited to date, regardless of plant transformation methodology (Lu et al. 2018; Rato et al. 2021; Tyagi et al. 2020). Therefore, much work remains to optimize these systems across economically important crop plants, since proof-of-concept in most of these studies was demonstrated in *N. benthamiana* or Arabidopsis.

Given the success of the 40+ plant viruses engineered as VIGS or protein expression vectors, solving current limitations of viral CRISPR/Cas9 vectors will undoubtedly yield remarkable results in plant genome editing studies (Cody and Scholthof 2019; Wang et al. 2020). Plant species- or cultivar-specific resistance to VIGE vectors are increasingly less problematic as the number of available vectors grows. The current bottleneck of many VIGE vector systems for generating stress-resistant crops is the low to no recovery of plants with heritable gene edits (Wang et al. 2020). Many researchers have discovered during VIGE system development that despite robust somatic cell genome editing, few progeny plants inherited the edits, indicating virus exclusion from the germline meristematic cells (Table 1). The totipotency of plant cells allows regeneration of plants from edited somatic tissue via technically time-consuming and fastidious culturing methods, which provides a mechanism for recovering edited plants similar to that of traditional CRISPR/Cas9 delivery methods (Atkins and Voytas 2020). Although plant regeneration is an advancement over transient VIGS assays, VIGE vector systems will likely gain popularity only once germline edits are reliably obtained to avoid the long timelines of traditional plant transformation methods. To overcome this current pitfall, increased virus or gRNA distribution within the plant could boost the number of inherited genome edits. As discussed earlier, work by several groups showed that fusing gRNAs with mobile RNAs such as tRNA or *FT* RNA sequences greatly increased the likelihood of heritable editing (Ellison et al. 2020; Lei et al. 2021; Li et al. 2021; Uranga et al. 2021a).

In addition to effective germline targeting in a given plant/virus system, production of a desired phenotype such as drought tolerance or pathogen resistance requires identification of appropriate gene targets. Due to the robustness of viral infection, a positive attribute of viral delivery of CRISPR/Cas9 constructs is that the phenotypic outcome of gene editing can be analyzed directly in infected tissue prior to seed set and plant selection, enabling timely refinement or modification of the gRNA sequences if needed for optimal results (Cody and Scholthof 2019). This facilitates the identification of appropriate gene targets from a list of candidates, for example. Recent work with traditional non-viral CRISPR/Cas9 assays have targeted a number of genes impacting plant responses to drought, heat stress, salinity, and disease (Das et al. 2018; Sun et al. 2021). Additionally, the traditional VIGS systems have been successfully used to study plant responses to both biotic and abiotic stresses (Dommes et al. 2019; Dulermo et al. 2009; Shi et al. 2021). Therefore, it is only a matter of time before VIGE systems will be employed to similarly study gene function during pathogen infection or under abiotic stress. Indeed, as discussed below, the expanding toolbox of CRISPR/Cas9 derivatives paired with VIGE vectors will allow more thorough analysis of stress responses than by the traditional methods alone.

**Fig. 1:**
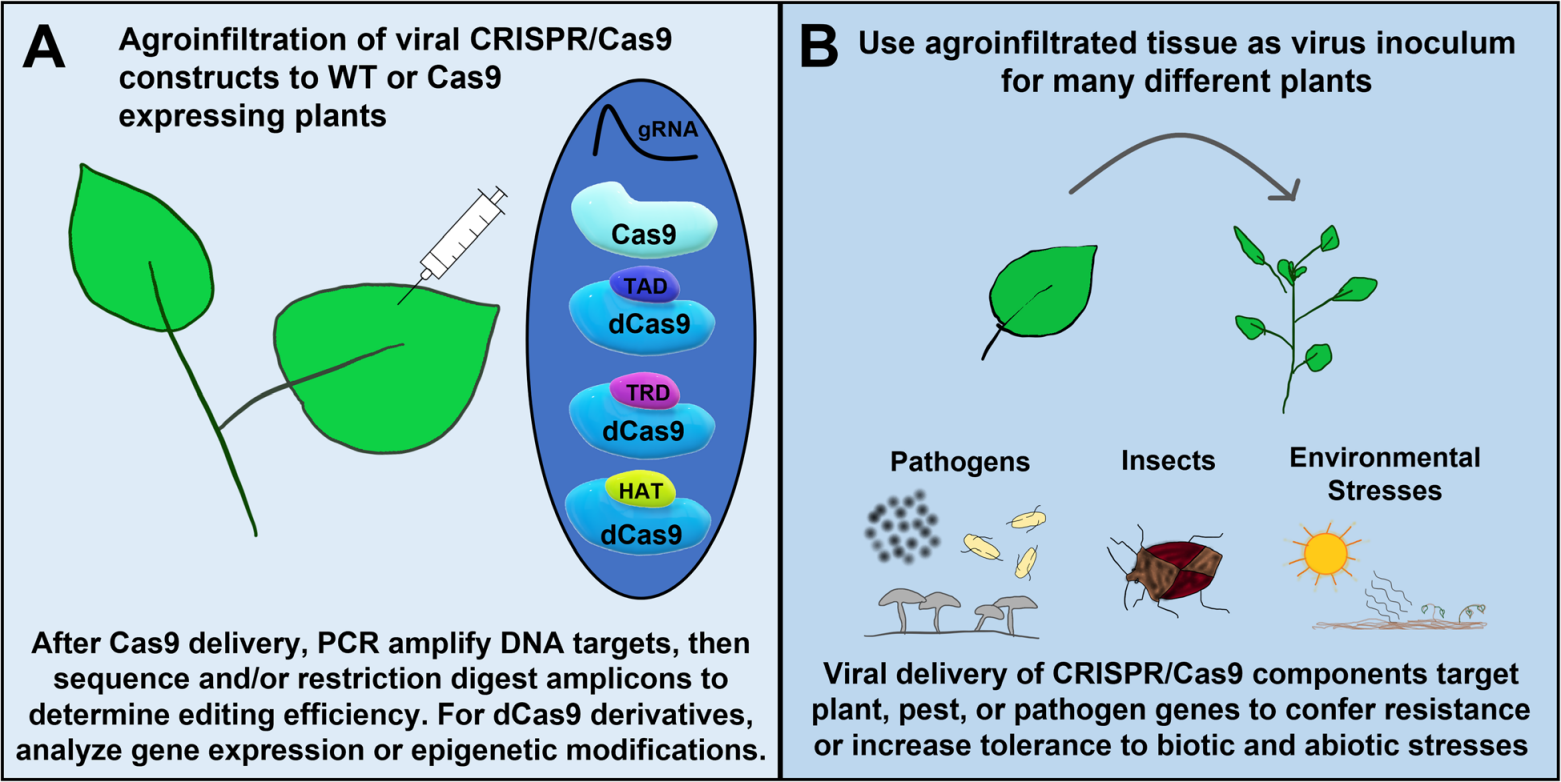
Applications for viral delivery of CRISPR/Cas9 components to plants. **A**. Agroinfiltration of viral constructs to model plant species such as *N. benthamiana* (wild type (WT) or Cas9-expressing) is useful for characterization of the system as well as generating inoculum for other plants less amenable to agroinfiltration. In addition to genomic editing by Cas9, deactivated forms (dCas9) fused with transcriptional activation (TAD) or repression (TRD) domains are available to study effects of gene expression modulation. Further dCas9 derivative fusions with epigenetic modifiers have recently been developed, such as fusion with a histone acetyltransferase (HAT). **B**. The versatility of viral CRISPR/Cas9 delivery to plants allows tunable studies targeting plant and pest genes to increase resistance to biotic and abiotic stresses

Broad application of virus delivery of CRISPR/Cas9 constructs will aid researchers much like VIGS systems have greatly advanced our understanding of plant gene function (Fig. 1). Additionally, viral CRISPR/Cas9 systems can be further modified to include Cas9 derivatives such as dCas9 fusions with transcriptional activators or repressors to further examine plant gene function, as is described for the recently published VipariNama rapid phenotyping system with TRV (Khakhar et al. 2021). Also, dCas9 fusions with epigenetic modifiers such as histone deacetylases or acetyltransferases have been reported for animal systems, opening up new opportunities for plant epigenetic studies (Kwon et al. 2017; Nakamura et al. 2021; Roca Paixão et al. 2019). Indeed, it was recently shown that TRV delivery of gRNAs to Arabidopsis expressing dCas9-TET1 – a human DNA demethylase - resulted in a heritable reduction of FLOWERING WAGENINGEN promoter methylation (Ghoshal et al. 2020). In addition to Cas9-based editing tools, viral delivery of alternate CRISPR/Cas complexes are also under development for gene editing. A recent report illustrated the use of the comparatively smaller Cas12a nuclease in tobacco etch virus (TEV) co-infiltrated with potato virus X (PVX) carrying an *FT* gRNA in *N. benthamiana* (Uranga et al. 2021b). Interestingly, this compatible interaction between the two viruses resulted in a nearly 75% editing efficiency (Uranga et al. 2021b). The Cas13 nuclease can target single stranded RNA, rather than double stranded DNA targeted by Cas9, leading to interest in applications targeting RNA viruses (Cao et al. 2021; Wolter and Puchta 2018; Yu et al. 2021). Additionally, deactivated Cas13 (dCas13) can be used for a variety of interesting applications, including RNA virus detection by binding fluorescently-tagged dCas13 to the RNA target (Wolter and Puchta 2018).

As highlighted in this review, many new CRISPR/Cas9 viral delivery systems are being developed and are ready for application in plant functional genomics studies. Ultimately, once biosafety and bioethics concerns have been addressed, it may eventually be possible to deploy these virus constructs via their natural insect vectors to rapidly save stressed crop plants in field applications. Thus, we anticipate these technologies will be adopted by many laboratories to advance their crop improvement studies for understanding gene function and developing disease/insect resistant and abiotic stress tolerant cultivars.

## Data Availability

Not applicable.
